# Road Lane Detection by Discriminating Dashed and Solid Road Lanes Using a Visible Light Camera Sensor

**DOI:** 10.3390/s16081313

**Published:** 2016-08-18

**Authors:** Toan Minh Hoang, Hyung Gil Hong, Husan Vokhidov, Kang Ryoung Park

**Affiliations:** Division of Electronics and Electrical Engineering, Dongguk University, 30 Pildong-ro 1-gil, Jung-gu, Seoul 100-715, Korea; hoangminhtoan@dongguk.edu (T.M.H.); hell@dongguk.edu (H.G.H.); vokhidovhusan@dongguk.edu (H.V.)

**Keywords:** road lane detection, left and right boundaries of road lane, dashed and solid road lanes, autonomous vehicles

## Abstract

With the increasing need for road lane detection used in lane departure warning systems and autonomous vehicles, many studies have been conducted to turn road lane detection into a virtual assistant to improve driving safety and reduce car accidents. Most of the previous research approaches detect the central line of a road lane and not the accurate left and right boundaries of the lane. In addition, they do not discriminate between dashed and solid lanes when detecting the road lanes. However, this discrimination is necessary for the safety of autonomous vehicles and the safety of vehicles driven by human drivers. To overcome these problems, we propose a method for road lane detection that distinguishes between dashed and solid lanes. Experimental results with the Caltech open database showed that our method outperforms conventional methods.

## 1. Introduction

Accurate detection of road lanes is an important issue in lane departure warning systems and driver assistance systems. Detecting lane boundaries enables vehicles to avoid collisions and issue a warning if a vehicle passes a lane boundary. However, lane boundaries are not always clearly visible. This can be caused, for instance, by poor road conditions, insufficient quantity of paint used for marking the lane boundary, environmental effects (e.g., shadows from objects like trees or other vehicles), or illumination conditions (street lights, daytime and nighttime conditions, or fog). These factors make it difficult to discriminate a road lane from the background in a captured image. To deal with these problems, current research applies various methods ranging from low-level morphological operations to probabilistic grouping and B-snakes [1,2,3]. Detail explanations of previous works are shown in Section 2.

## 2. Related Works

The methods for lane departure warning can be classified into two categories: sensor-based methods and vision-based methods. Sensor-based methods use devices such as radar, laser sensors, and even global positioning systems (GPS) to detect whether a vehicle departed a lane based on the information of the vehicle ahead or the position calculated by GPS. These devices can also be used for obstacle detection. Their main advantage is their scanning distance (up to 100 m) and their high reliability in dust, snow, and other poor weather conditions. However, these methods cannot accurately detect the lane positions, and the information they provide is unreliable inside a tunnel or if no other vehicle is ahead. Therefore, most of the recent research approaches have been focusing on developing vision-based solutions and using additional sensors to enhance the results.

The vision-based methods detect road lanes based on the features of a camera image such as color gradient, histogram, or edge. We can divide vision-based solutions into two main classes. One is the model-based methods [1,2,3,4,5,6,7,8,9,10,11,12,13,14,15,16,17], which create a mathematical model of the road structure. They use the geometric coordinates of the camera and the road as input parameters and depend on their accuracy. To determine the parameters, the initial configuration information is merged with feature points of the lane markings taken from an image of the road. For example, Xu et al. used a B-spline based road model to fit the lane markings and a maximum deviation of position shift method for identifying the road model’s control points [1]. Li et al. used an extended Kalman filter in addition to a B-spline curves model to guarantee a continuous lane detection [2]. Tan et al. focused on detecting a curve lane using improved river flow and random sample consensus (RANSAC) under challenging conditions based on a hyperbola-pair lane model [5]. Zhou et al. presented a lane detection method based on a geometrical model of the lane and Gabor filter [6]. In earlier work [13], they had proposed a lane detection method that used gradient-enhancing conversion to guarantee an illuminating-robust performance. In addition, they used an adaptive canny edge detector, a Hough transformation (HT), and a quadratic curve model. Li et al. employed an inverse perspective mapping (IPM) model [14] to detect a straight line in an image. Chiu et al. introduced a lane detection method using color segmentation, thresholding, and fitting with a quadratic function model [15]. Mu et al. determined candidate regions of lane markings by object segmentation, applied a sober operator to extract redundancy edges, and used piecewise fitting with a linear or parabolic model to detect lane markers [17]. With model-based methods, lane detection becomes a problem of solving mathematical models. The accuracy of the detection depends not only on the initial input parameters of the camera or the shape of the road but also on the feature points extracted from a captured image of the road.

The other class of vision-based methods are the feature-based methods [18,19,20,21,22,23], which can discriminate feature points of lane markings from the non-lane areas by characteristic features of the road, such as color, gradient, or edge. Chang et al. applied a canny edge detector to investigate boundaries and proposed an edge-pair scanning method and HT to verify that the edges belonged to lane markings [18]. In previous research [19], they had introduced a method for determining the adaptive road region-of-interest (ROI) and locating the road lane. Chen et al. proposed a method to detect a lane with a downward looking color camera and a binormalized adjustable temple correlation method [20]. Benligiray et al. suggest detecting lanes by detecting line segments based on the EDLines algorithm [21]. In previous research [22], they had detected lanes using canny edge detector and HT based on vanishing points. Sohn et al. proposed an illumination invariant lane detection algorithm using ROI generation based on vanishing point detection and lane clustering [23]. The feature-based methods are simple, but they require a clear and strong color contrast of the lane and good road conditions with little changes in the surrounding environment. Most of the previous (model-based and feature-based) methods detect the central line of the road lane and do not locate the accurate left and right boundaries of the road lane. In particular, they do not discriminate the dashed and solid lanes when detecting the road lanes. In previous research [24], they classified lanes based on a linear–parabolic lane model, an automatic on-the-fly camera calibration, an adaptive smoothing scheme, pairs of local maxima–minima of the gradient, and a Bayesian classifier using mixtures of Gaussians. Although their method can classify the kinds of lane such as dashed, solid, dashed solid, solid dashed, and double solid ones, their method did not detect the starting and ending positions of lane. That is, with the lane region within ROI of image, their method classified only the kinds of lane without detecting the exact starting and ending points. That is because the correct classification based on Bayesian classifier can be possible even with a little (detection) error of starting and ending points, and the little amount of error can be compensated by the Bayesian classifier. Therefore, in their research [24], they did not show the accuracy of detecting the starting and ending points, but showed only the classification error of five kinds of road lane.

Different from their method, we correctly detect the starting and ending positions of lane with the discrimination dashed and solid lanes. By studying pros and cons of the existing research approaches, we decided to use a feature-based lane detection method and to detect a lane’s accurate left and right boundaries by discriminating the dashed and solid lanes. Our research is novel in the following four aspects compared to other work.

- In most previous researches, they detect only the centerline of the left and right boundaries of a road lane. Different from them, our method can detect the accurate left and right boundaries of a road lane.
- In most previous studies, they detected the starting and ending positions of a road lane without discriminating the dashed and solid ones. In some research, they just classified the kinds of road lane such as dashed, solid, dashed solid, solid dashed, and double solid ones without detecting the starting and ending positions of a road lane. Different from them, our method correctly detects the starting and ending positions of lane with the discrimination of dashed and solid lanes.
- We can remove incorrect line segments using the line segments’ angles and merging the line segments according to their inter-distance. In order to detect curve lane, the angular condition is adaptively changed within the upper area of ROI based on tracing information of angular changes of line segments.
- Using a perspective camera model, the adaptive threshold is determined to measure the distance and used to detect the final line segments of the road lane’s left and right boundaries.

Table 1 presents a summary of our comparison of existing research on lane detection and our proposed method.

The remainder of this paper is organized as follows. We provide an overview of the proposed method and an algorithm for road lane detection in Section 3. Section 4 discusses the experimental results, and Section 5 presents the conclusions.

## 3. Proposed Method

### 3.1. Proposed Method

**Figure 1:**
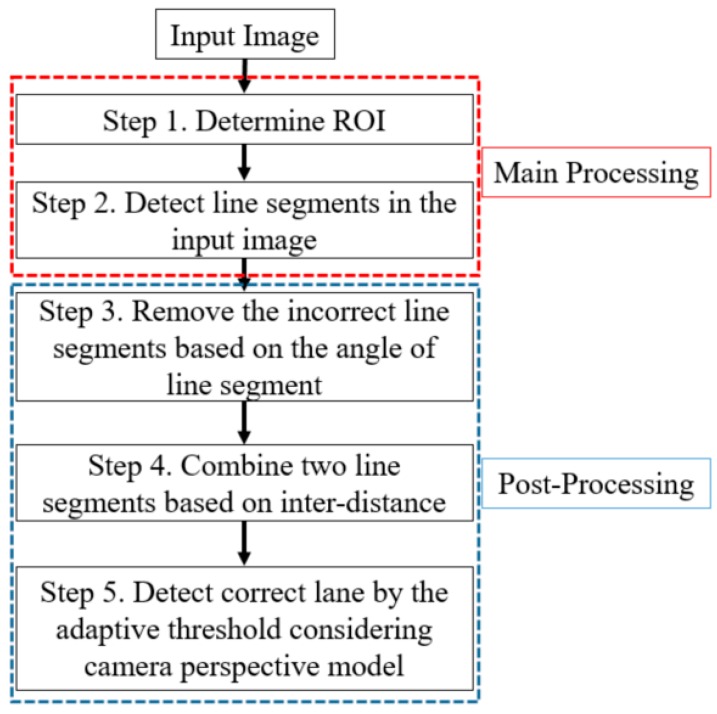
Overall procedure of the proposed method.

An overview of the proposed method is presented in Figure 1.

Figure 1 depicts the whole procedure of our proposed method. In our lane detection system, the algorithm has two main stages, main processing (Step 1 and Step 2) and post-processing (Step 3 to Step 5). In the first step, we define the ROI of the captured image and locate the line segments in the ROI. Then, we remove incorrect line segments based on the angles of the line segments and by merging them according to their inter-distance. Finally, using the perspective camera model, we determine the adaptive threshold to measure the distance and use it to detect the final line segments of the left and right boundaries of the road lane.

### 3.2. Determination of ROI

**Figure 2:**
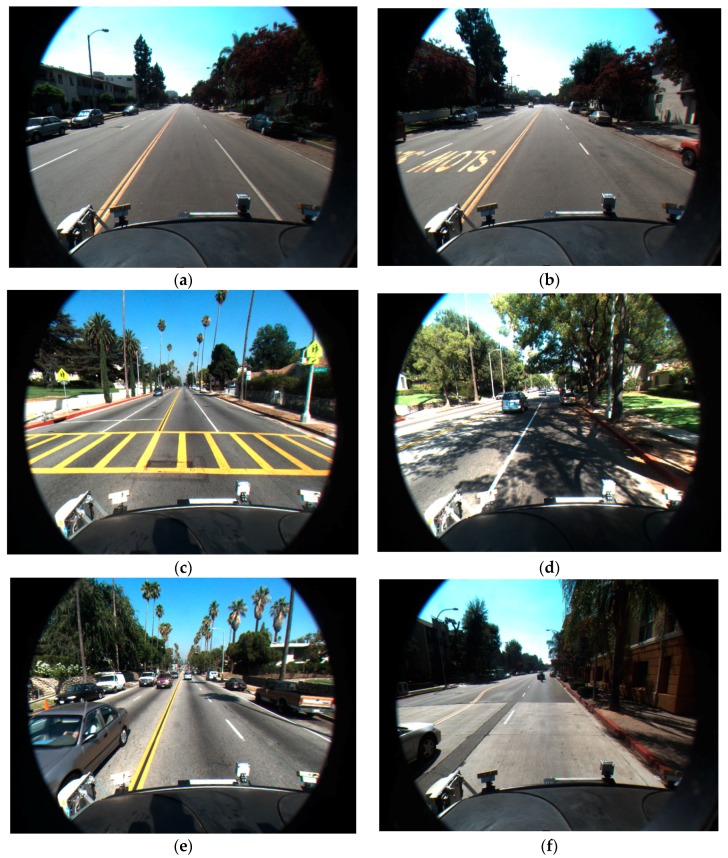
Examples of input images: (**a**) input image without road markings, crosswalk and shadow; (**b**) input image including road markings; (**c**) input image including crosswalk; and (**d**–**f**) input images including shadow.

**Figure 3:**
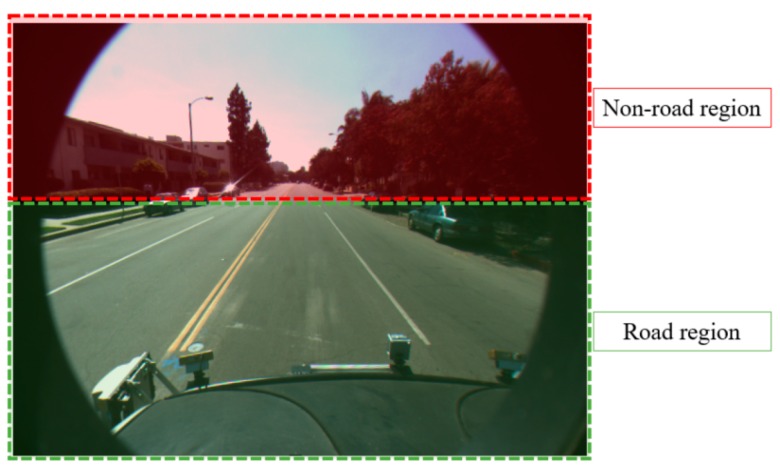
Region without or with road.

**Figure 4:**
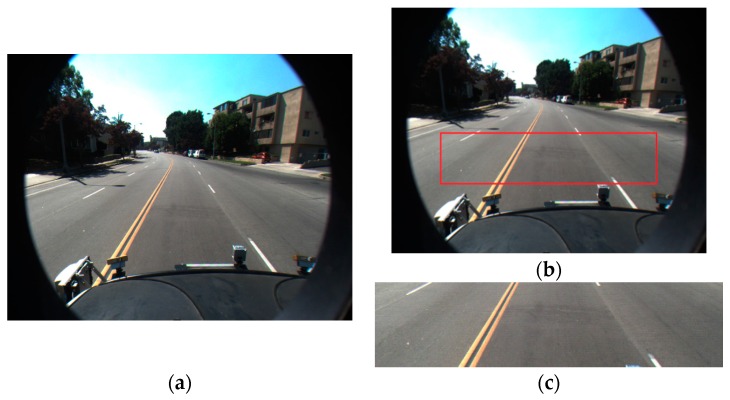
Defining the region-of-interest (ROI) in input image: (**a**) input image; (**b**) ROI; and (**c**) ROI image.

Defining the ROI in the captured image gives us two advantages. First, lanes always appear within the predetermined region of the image when the position and direction of the camera are fixed. This is shown in Figure 2, Figure 3 and Figure 4. Therefore, we do not need to perform lane detection in the whole image, but only in the restricted area. If the lane detection is done only in the ROI and not in the whole image, the effect of environmental noises such as rain, fog, or poor weather conditions can be lessened. In addition, the complexity and computational time of the lane detection can be significantly reduced.

Previous research defined the ROI based on vanishing points [13]. However, this takes a lot of processing time and might determine an incorrect ROI if the vanishing points were incorrect. Our research is mainly focused on detecting the starting and ending positions of straight and curve lanes with the discrimination of dashed and solid lanes within the ROI. Therefore, in our research, we do not automatically estimate ROI, but use the predetermined ROI shown in the image (the ROI of red box in Figure 4b), for lane detection. In our experiments, we used two kinds of open databases such as Caltech and SLD datasets (see details in Section 4). All images have the size of 640 × 480 pixels. Based on these dimensions, with Caltech dataset, the left-upper position (x and y coordinates) of ROI is determined as (100, 245), and the width and height of the ROI are empirically determined as 440 and 100 pixels. With SLD dataset, the left-upper position (x and y coordinates) of ROI is determined as (120, 320), and the width and height of the ROI are also empirically determined as 440 and 100 pixels. In the case of using images of different size, the left-upper position, width, and height of ROI are proportionally changed based on the width and height of the original image.

### 3.3. Lane Detection by Locating Line Segments

**Figure 5:**
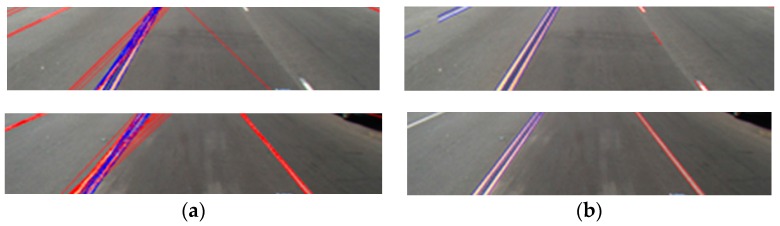
Comparisons of line detection: (**a**) line detection using conventional edge detector and Hough transformation (HT); and (**b**) line extraction using line segment detector (LSD).

Some of the existing research approaches convert an input image into an IPM or bird’s eye view image [10,11,12,14,25] to represent lanes as vertical and parallel in the image. However, in some cases, the lanes cannot be vertical and parallel because we would need to adjust the set of parameters for obtaining the IPM or bird’s eye view image according to the relative position of the lane to the camera. The position of a vehicle and its camera can slightly change between two road lanes. Moreover, the camera parameters need to be known in advance to obtain an accurate image by the IPM or bird’s eye view image. In other research approaches, lanes were detected using HT [2,8,13,14,15,18,19,22,23]. However, it takes a long processing time and detects too many incorrect line segments, as shown in Figure 5a. In addition, it cannot detect a lane by discriminating between the dashed and solid lanes.

To solve these problems, we use a line segment detector (LSD) [26,27] for locating the line segments in an image. The LSD method is supposed to work on any digital image without depending on parameter tunning. The LSD algorithm controls the number of false detections based on previous research [28], and uses a contrario validation method based on Desolneux and coworker’s research [29,30]. Let *S* = $\left\{s_{1},\;s_{2},\;\ldots ,\;s_{k}\right\}$ be the set of line segments extracted from an ROI image using the LSD algorithm. Each line segment $s_{i},$ (i=1, 2, …,k) is defined as.

(1) $$s_{i}=\left\{x_{1i},\;y_{1i},\;x_{2i},\;y_{2i},\theta_{i}\right\},\;\left(i=1,\;2,\;\ldots ,k\right)$$

where $\left(x_{1i},\;y_{1i}\right)$ and $\left(x_{2i},\;y_{2i}\right)$ are the coordinates of the starting point and the ending point of line segment $s_{i}$, respectively. $\theta_{i}$ is the angle of line segment $s_{i}$ and is calculated by Equation (2).

(2) $$\theta_{i}\;=\frac{180}{\pi }\arctan\left(\frac{y_{2i}-y_{1i}}{x_{2i}-x_{1i}}\right),\;\left(i=1,\;2,\;\ldots ,\;k\right)$$

As shown in Figure 5, the LSD method can detect more correct line segments than the HT method.

We did not quantitatively compare the performance of line segment detection by LSD and HT. Instead, with some images of Caltech database and SLD database (used in our experiments of Section 4), we checked the performance. We used the already published LSD method. However, the main contributions of our research are not LSD method but the post-processing in order to detect the accurate starting and ending points of straight and curve lanes with the discrimination of the broken (dashed) and unbroken (solid) lanes as shown in Section 3.4.

### 3.4. Correct Lane Detection Based on Post-Processing

We remove the line segments that were detected incorrectly and locate the accurate line segments based on the features of the road lane, such as angle and inter-distance between left and right boundaries of the lane.

#### 3.4.1. Eliminating Incorrect Line Segment Based on Angle

**Figure 6:**
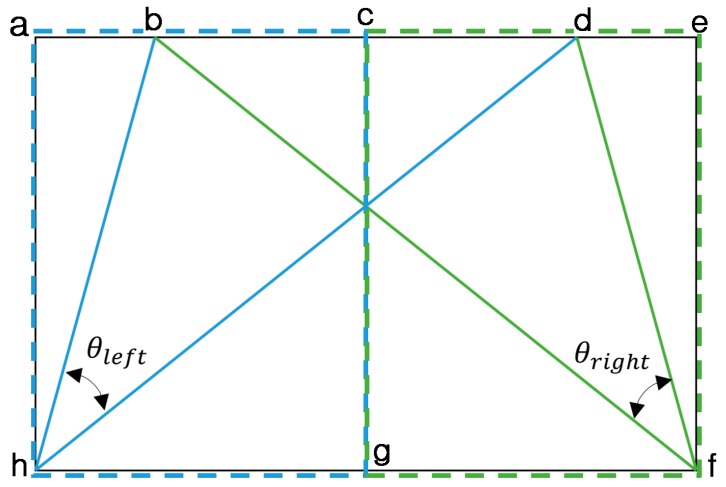
Angle feature used for eliminating incorrect line segments.

As Figure 5b illustrates, the LSD algorithm also detects many incorrect line segments. We need to eliminate the incorrect line segments to reduce the computational time and complexity. In our research, we first use the angle of a road lane to eliminate the incorrect line segments. In Figure 6, the left side (the rectangular region of **acgh**) and right side (the rectangular region of **cefg**) are divided, and two angular ranges ($\theta_{left}\;\left(25^{0}\;\sim \;75^{0}\right)$ and $\theta_{right\;}(105^{0}\;\sim \;155^{0}$)) for the correct line segments are determined on each side, as shown in Equations (3) and (4). The same parameters of angular ranges of Equations (3) and (4) are used in two open databases of our experiments in Section 4. Because vehicle including camera usually moves between left and right road lanes, the left and right road lanes can be estimated to be included in the areas of triangles (**bdh**) and (**bdf**) of Figure 6, respectively. Here, $\theta_{left}$ defines the angular area between two lines of **bh** and **dh** of Figure 6. $\theta_{right}$ defines the angular area between two lines of **bf** and **df** of Figure 6.

(3) $$\begin{array}{l} S_{\text{left}}=\{s_{i}^{L}\;|x_{1i}\leq \frac{w_{ROI}}{2}-1,\;\theta_{i}^{L}\in \left[25^{0},\;75^{0}\right]\}\;,\;\left(i=1,\;2,\;\ldots ,\;p\right)\left(if\;"H_{ROI}/3\;\leq \;y_{1i}\leq \;H_{ROI}-1"\right)\\ S_{\text{left}}=\{s_{i}^{L}\;|x_{1i}\leq \frac{w_{ROI}}{2}-1,\theta_{i}^{L}\in \left[\;\theta_{i}^{L*}-10^{0}\leq \;\theta_{i}^{L}\leq \;\theta_{i}^{L*}+10^{0}\right]\}\;,\;\\ \left(i=1,\;2,\;\ldots ,\;p\right)\;(else\;if\text{ “}0\;\leq \;y_{1i}\;<\;H_{ROI}/3") \end{array}$$

(4) $$\begin{array}{l} S_{\text{right}}=\{s_{i}^{R}\;|x_{1i}>\frac{w_{ROI}}{2},\;\theta_{i}^{R}\in \left[105^{0},\;155^{0}\right]\}\;,\;\;\left(i=1,\;2,\;\ldots ,\;q\right)\;\left(if\;"H_{ROI}/3\;\leq \;y_{1i}\leq H_{ROI}-1"\right)\\ S_{\text{right}}=\{s_{i}^{R}\;|x_{1i}>\frac{w_{ROI}}{2},\;\theta_{i}^{R}\in \left[\;\theta_{i}^{R*}-10^{0}\leq \;\theta_{i}^{R}\;\leq \;\theta_{i}^{R*}+10^{0}\right]\}\;,\left(i=1,\;2,\;\ldots ,\;q\right)\;(else\;if\;“0\;\leq \;y_{1i}\;<\;H_{ROI}/3") \end{array}$$

where $x_{1i}$ is the *x* coordinate of the starting point of line segment $s_{i}$ in Equation (1), and $\theta_{i}$ is the angle of line segment $s_{i}$ in Equation (2). In addition, $W_{ROI}$ is the width of the ROI region (the distance between **a** and **e** (or **h** and **f**) of Figure 6). As explained in Section 3.2, $W_{ROI}$ is 440 pixels in our research. $H_{ROI}$ is the height of the ROI region (the distance between **a** and **h** (or **e** and **f**) of Figure 6). As explained in Section 3.2, $H_{ROI}$ is 100 pixels in our research.

**Figure 7:**
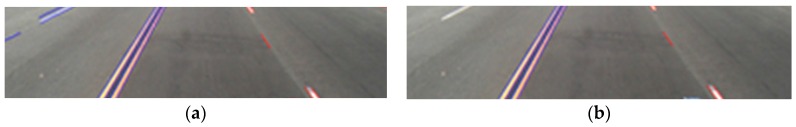
Removing the incorrect line segments based on angle features: (**a**) image including the detected line segments; and (**b**) image with the incorrect line segments removed.

Each line segment has two positions, namely its starting and ending positions. In our research, we consider the higher position (i.e., the position with a lower y coordinate) to be the starting point in the image with the origin (0, 0) defined as the left-upper most corner. All line segments whose starting point has an x-coordinate between 0 and $W_{ROI}$/2-1 are considered to belong to the left side (the rectangular region of **acgh** of Figure 6) as shown in Equation (3). All other line segments belong to the right side (the rectangular region of **cefg** of Figure 6) as shown in Equation (4). That is, the sets of line segments ($s_{i}^{L}$ and $s_{i}^{R}$) satisfying the conditions of Equations (3) and (4) are obtained as S_left_ and S_right_, respectively, and are considered correct line segments. Figure 7 shows the example where the incorrect line segments are removed based on angle features.

As explained at the end of this Section, the conditions of Equations (3) and (4) are divided into two parts ($if\;"H_{ROI}/3\;\leq \;y_{1i}\leq \;H_{ROI}-1"$ and *else if* “$0\;\leq \;y_{1i}\;<\;H_{ROI}/3"))$ according to $y_{1i}$ (the *y* coordinate of the starting point of line segment $s_{i}$ in Equation (1)) in order to solve the detection problem of curve lane. Detail explanations are shown at the end of this Section.

Figure 6 denotes the angular range of the correct line segments from a camera view. All valid line segments should lie within these two areas, which are defined by the angular ranges of $\theta_{left}$ (the angular area between two lines of **bh** and **dh**) and $\theta_{right}$ (the angular area between two lines of **bf** and **df**). We consider the line segments outside these areas as incorrect and are eliminated them.

As shown in [31,32], the curve lane having severe curvature is usually observed only in the upper area of ROI. Therefore, our method uses the 2nd ones of Equations (3) and (4) (condition of *else if* “$0\;\leq \;y_{1i}\;<\;H_{ROI}/3"$) with Figure 6 within only the upper area of the ROI (where the curve lane having severe curvature can be observed). That is, because the width and height of ROI are, respectively, 440 and 100 pixels in our research (as explained in Section 3.2), the upper-left and lower-right positions of upper area in the ROI are (0, 0) and (439, 33 (100/3)).

In this upper area, the angular ranges of $\theta_{right}$ and $\theta_{left}$ are adaptively changed as shown in the 2nd ones of Equations (3) and (4) (condition of *else if* “$0\;\leq \;y_{1i}\;<\;H_{ROI}/3"$). That is, based on tracing information ($\theta_{i}^{L*}$ and $\theta_{i}^{R*}$ of the 2nd ones of Equations (3) and (4)) of the angular of previous line segment (whose position is immediately below, but the closest to the current line segment to be traced), our method adaptively changes the angular range ($\theta_{i}^{L}$ and $\theta_{i}^{R}$) in this upper area as shown in “$\theta_{i}^{L}\in \left[\;\theta_{i}^{L*}-10^{0}\leq \;\theta_{i}^{L}\;\leq \;\theta_{i}^{L*}+10^{0}\right]"\;\text{and}\;\theta_{i}^{R}\in \left[\;\theta_{i}^{R*}-10^{0}\leq \;\theta_{i}^{R}\;\leq \;\theta_{i}^{R*}+10^{0}\right]$ of the 2nd ones of Equations (3) and (4).

Based on this, our method can correctly detect curve lane. In addition, through the line combination algorithm of Section 3.4.2, the pieces of line segments from a curve line can be correctly combined as a curve line.

#### 3.4.2. Combining the Pieces of Line Segments

Due to the impact of shadows, illumination changes, or incorrect detection of line segments, we can detect multiple line segments from one boundary of road lanes. Our system checks the conditions to determine whether two or more line segments should be combined into one longer line segment.

**Figure 8:**
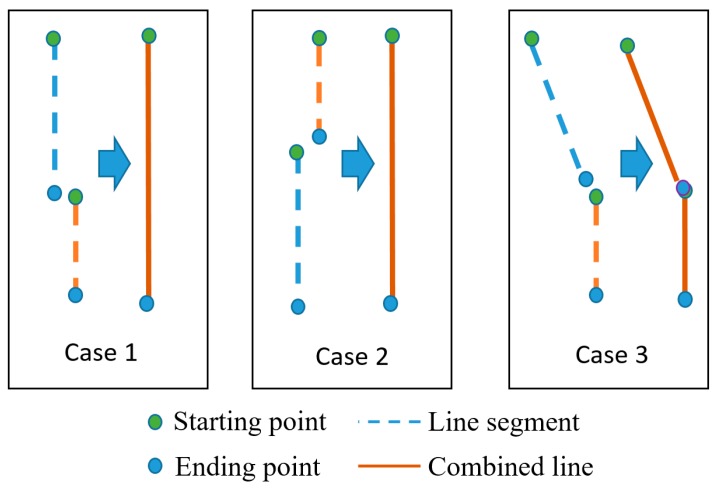
Angle feature used for eliminating incorrect line segments.

Figure 8 shows three cases where two or more line segments are combined into one longer one. The first two cases make one straight line whereas the last one makes a curve line. As mentioned in the previous section, we assume that the starting point of a line segment has a lower y-coordinate than the ending point. The dashed lines in Figure 8 denote line segments, whereas the solid lines represent the merged line segment. Blue and green circles represent the starting point and ending point, respectively.

To evaluate the connectivity of two line segments, the following two conditions should be satisfied based on the distance threshold (*thr_dst_*) and the angle threshold (*thr_angle_*). The thresholds were obtained, empirically. In our research, *thr_dst_* and *thr_angle_* are 3 pixels and 2 degrees, respectively, on both Caltech and SLD databases which were used in our experiments (see details in Section 4). However, *thr_dst_* is proportionally changed based on the width and height of the original image.

We assume that the Euclidean distance between the starting point of the *i*th line and the ending point of the *j*th line, or the ending point of the *i*th line and the starting point of the *j*th line is $diff_{dst}$. If $diff_{dst}$ is less than *thr_dst_* (the first condition), our system checks the second condition based on the angle in order to decide whether two line segments are part of a straight line or a curve line.

As explained in the previous section, our system can obtain the angle of each line segment and compare the angular difference of two line segments (called $diff_{angle}$) to a predefined threshold (*thr_angle_*). *Line H* is the new line that is obtained by combining the *i*th line with the *j*th line.

(5) $$Line\;H=\{\begin{array}{c} straight\;line,\;if\;diff_{angle}\leq thr_{angle}\;\left(\text{cases }1\text{ and }2\text{ of Figure }8\right)\\ curve\;line,\;otherwise\;\left(\text{case }3\text{ of Figure }8\right)\; \end{array}$$

The below Algorithm 1 provides more details. Rough explanations of this algorithm are as follows. If the Y starting position of line *I* is lower than that of line *J*, the distance between the ending position of line *I* and the starting one of line *J* is measured. If this distance is less than threshold (*thr_dst_*) and the angle between these two lines is less than threshold (*thr_angle_*), these two lines are combined as a new straight line (Case 1 of Figure 8). If the distance condition is satisfied, but the angle condition is not, these two lines are combined as a new curve line (Case 3 of Figure 8).

If the Y starting position of line *I* is higher than that of line *J*, the distance between the starting position of line *I* and the ending position of line *J* is measured. If this distance is less than threshold (*thr_dst_*) and the angle between these two lines is less than threshold (*thr_angle_*), these two lines are combined as a new straight line (Case 2 of Figure 8). If the distance condition is satisfied, but the angle condition is not, these two lines are combined as a new curve line (Case 3 of Figure 8).
**Algorithm 1.** Line Combination Algorithm.Input: Set of line segments SOutput: Set of combined lines  While (line I ∈ S) {  Get starting point $I_{s},$ ending point $I_{e}$, and angle $I_{a}$
  While ((line J ≠line I) and (J ∈ S))  {   Get starting point $J_{s},$ ending point $J_{e},$ and angle $J_{a}$
   If ($I_{s}.y<J_{s}.y$)   {    $diff_{dst}$ = d($I_{e},\;J_{s})$    If ($diff_{dst}$ < thrdst)     {     $diff_{angle}$ = |$I_{a}$ – $\;J_{a}$|     If $(diff_{angle}$ <= $thr_{angle}$)     {      Define a new straight line K having $I_{s}$ and $J_{e}$ //Case 1 of Figure 8      Remove lines I and J     }     Else if $(diff_{angle}$ > $thr_{angle}$)     {      $I_{e}=J_{s}$      Define a new curve line having $I_{s}$, $J_{s}$, and $J_{e}$ //Case 3 of Figure 8     }    }  }  Else if ($I_{s}.y$ >= $J_{s}.y$)  {   $diff_{dst}$ = d($I_{s},\;J_{e})$   If ($diff_{dst}$ < thrdst)   {    $diff_{angle}$ = |$I_{a}$ – $\;J_{a}$|    If $(diff_{angle}$ <= $thr_{angle}$)    {     Define a new straight line K having $J_{s}$ and $I_{e}$ //Case 2 of Figure 8     Remove lines I and J    }    Else if $(diff_{angle}$ > $thr_{angle}$)    {     $I_{s}=J_{e}$     Define a new curve line having $J_{s}$, $I_{s}$, and $I_{e}$ //Case 3 of Figure 8    }   }  } }}

#### 3.4.3. Detecting the Left and Right Boundaries Based on Adaptive Threshold

Because of various factors such as varying illumination, shadows, and the abrasion of paint on the road lane, we can divide the detected road line into several discrete parts. “Line Combination Algorithm” (explained in Section 3.4.2) combines these parts into a line boundary, but further processing is necessary to detect more accurate lane boundaries.

**Figure 9:**
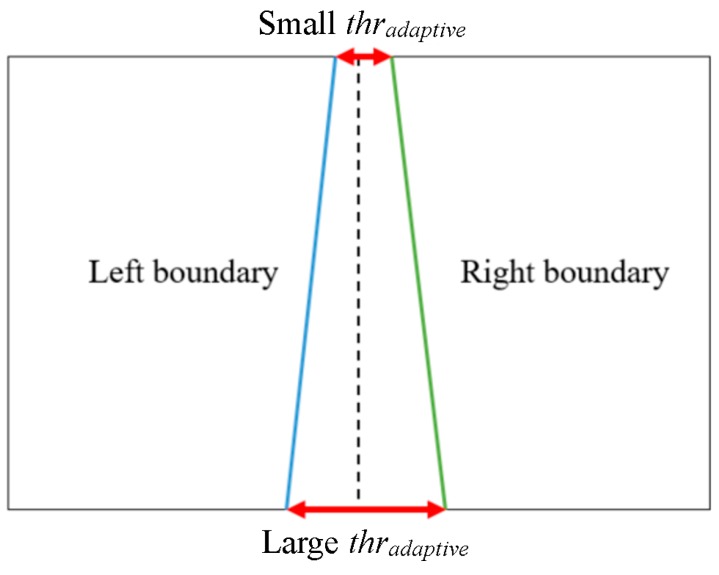
Distance between left and right boundaries of the road lane in the image.

A road lane always includes a left and a right edge boundary. If a detected line is on the left boundary, it has almost certainly a symmetrical counterpart on the right boundary and vice versa (Figure 9). From a camera view, the road lane appears as a trapezoid in the image as shown in Figure 9. This is because in a perspective camera model the further two points are away from the camera, the smaller their distance appears. Therefore, the adaptive threshold is determined by measuring the inter-distance between two starting points or ending points, as shown in Figure 9. Based on this threshold, we combine the two lines together. If the distance between the two starting positions and the distance between the two ending positions are less than the adaptive threshold (*thr_adaptive_* of Figure 9), the two lines are regarded as the correct left and right boundaries of the lane. In our research, *thr_adaptive_* has the range from 6 to 14 pixels. In the case of small *thr_adaptive_* in Figure 9, 6 pixels are used, whereas 14 pixels are used in the case of large *thr_adaptive_* in Figure 9. In the case of intermediate position between the upper and lower boundaries of Figure 9, the intermediate value by linear interpolation from 6 to 14 pixels is used as *thr_adaptive_* according to the Y position of line. Same parameter of *thr_adaptive_* is used in two open databases of our experiments in Section 4. However, *thr_adaptive_* is proportionally changed based on the width and height of the original image.

**Figure 10:**
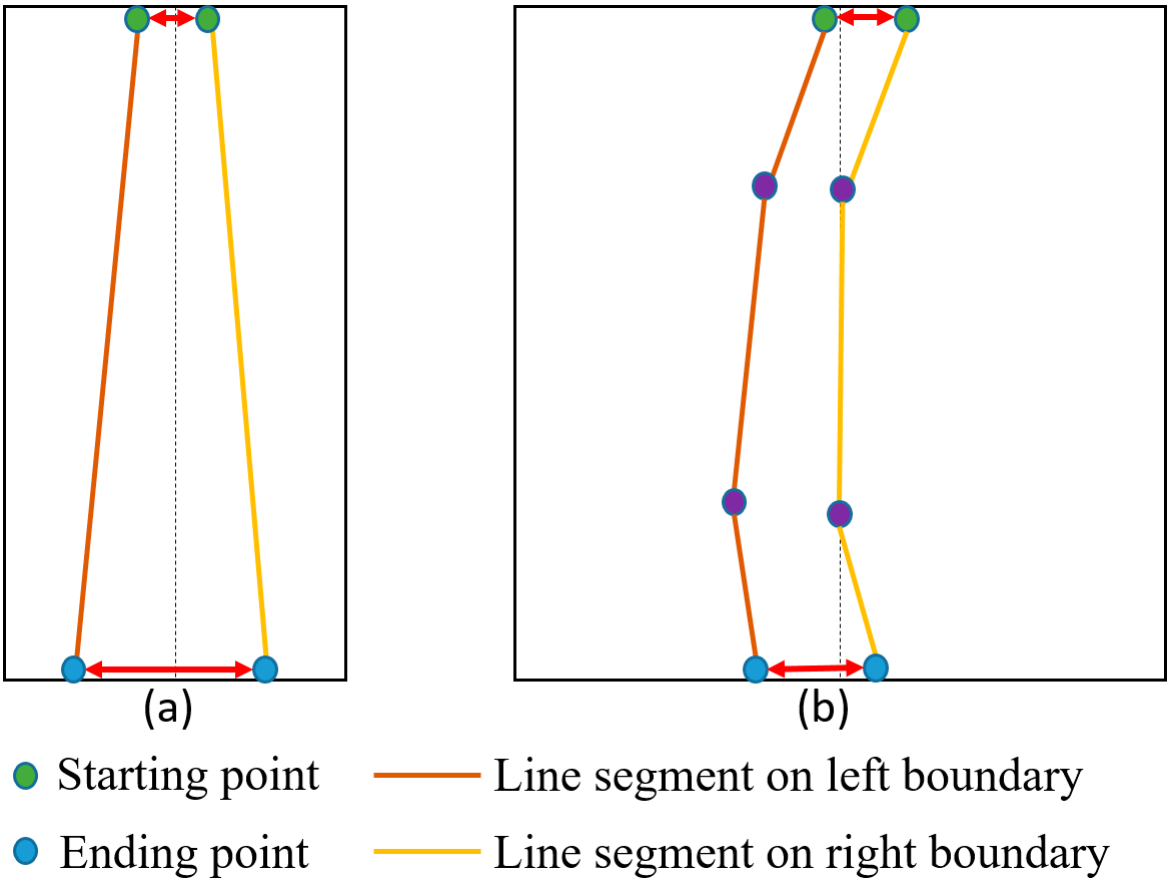
Distance between neighboring lines: (**a**) straight line; and (**b**) curve line.

However, in the case of a curve lane, we detect several line segments from the left and right boundaries as illustrated in Figure 10b. Consequently, we obtain several starting and ending positions for each line segment of the left and right boundaries. We select the two starting positions that have a y coordinate smaller than the other starting positions and the two ending positions that have a y coordinate larger than the other ending positions. We then calculate the distance between the two selected starting positions and the distance between the two selected ending positions. If the two distances are less than the adaptive threshold from the perspective camera model, we assume that we identified the correct left and right boundaries of the lane.

In our research, the curve line boundaries are also represented as the linear segments of small size. Because the length of line segment on curve line boundaries is small, the representation by linear segments as shown in Figure 10b produces small approximation error. The advantages of this representation are that we can reduce the processing time by representing the curve lane with the limited numbers of linear segments (not complicated polynomial curves).

## 4. Experimental Results

**Figure 11:**
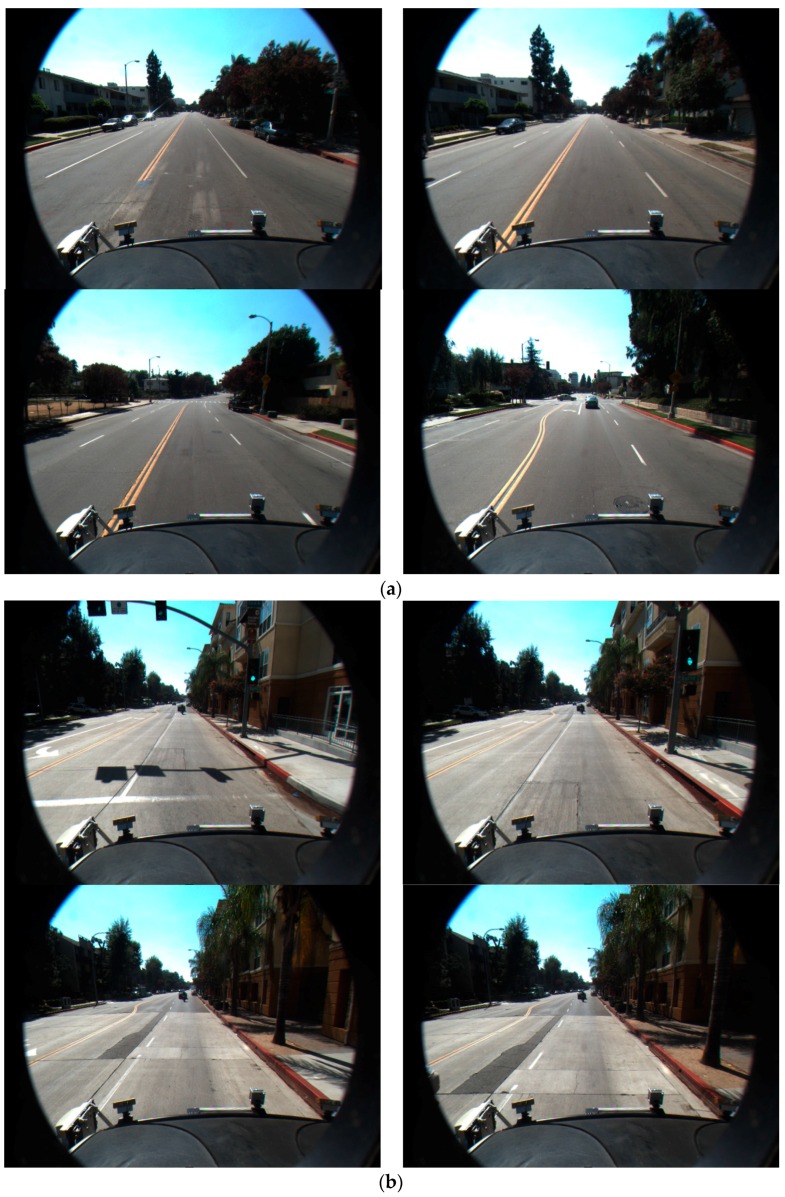

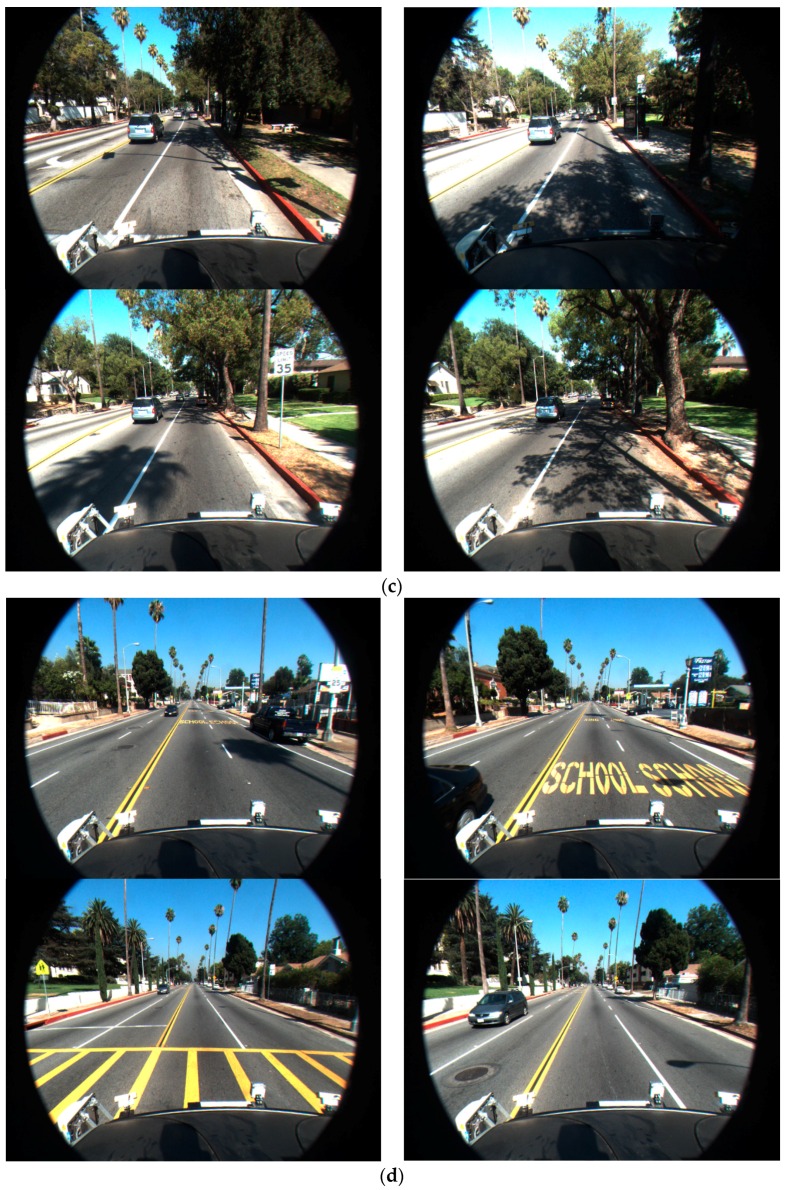
Examples of experimental datasets: (**a**) Cordova 1; (**b**) Cordova 2; (**c**) Washington 1; and (**d**) Washington 2.

We test our proposed method with the Caltech open database from [12], which consists of 866 total frames with an image size of 640 × 480 pixels. We implemented the proposed algorithm in Microsoft Visual Studio 2013 and OpenCV 3.0. Experiments were performed on a desktop computer with Intel Core™ i7 3.47 GHz (Intel Corporation, Santa Clara, CA, USA) and 12 GB memory (Samsung Electronics Co., Ltd., Suwon, Korea). Figure 11 shows the sample datasets used for the experiments.

To measure the accuracy of lane detection, the ground-truth (starting and ending) positions of lane were manually marked in the images. Because our method can detect the left and right boundary positions of a road lane by discriminating the dashed and solid lanes, all the ground-truth positions were manually marked to measure the accuracy. Based on the inter-distance between two starting positions (of ground-truth point and that detected by our method) and that between two ending positions (of ground-truth point and that detected by our method) of the lane, we determined whether the detection was successful or failed. If the two inter-distances are less than the threshold, the line detected by our method is determined as correct one. If not, it is determined as false one.

We define the correct lane point as positive data and the non-lane point as negative data. From that, we can define two kinds of errors, false positive (FP) errors and false negative (FN) errors. In addition, true positive (TP) is defined. Because we measure the accuracy only with the positive data and do not have any negative data (i.e., ground-truth non-lane point), true negative (TN) errors are 0% in our experiment. From that, we can obtain precision, recall, and F-measure [33,34]. The range of precision, recall, and F-measure is 0 to 1, with 0 being the lowest accuracy and 1 being the highest accuracy. In our evaluations, the numbers for TP, FP, and FN cases are represented as #TP, #FP, and #FN.

Table 2 shows the accuracies of our method. As indicated in Table 2, the precision, recall, and F-measure are about 0.90, 0.94, and 0.90, respectively. The road lines in Cordova 2 dataset are less distinctive compared to those in other datasets, which increases the FP detection by our method and decreases the consequent precision. The Washington 2 dataset includes more symbol markings (such as indicating words, crosswalk as shown in Figure 11d) on the road than other datasets. These symbol markings cause FP detection by our method, which decreases the consequent precision. Based on the results from Table 2, we can conclude that the proposed method works well with the images captured in various environments.

**Figure 12:**
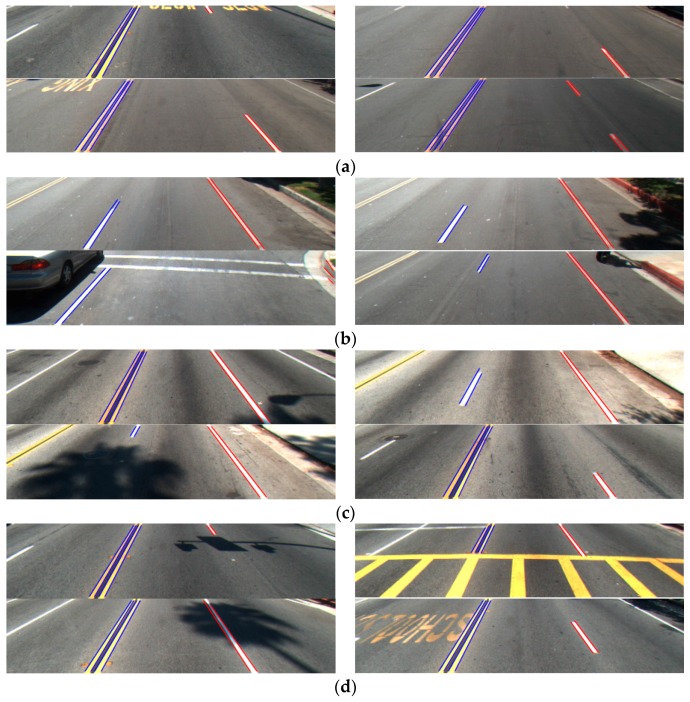
Examples of correct lane detection: (**a**) Cordova 1; (**b**) Cordova 2; (**c**) Washington 1; and (**d**) Washington 2.

**Figure 13:**
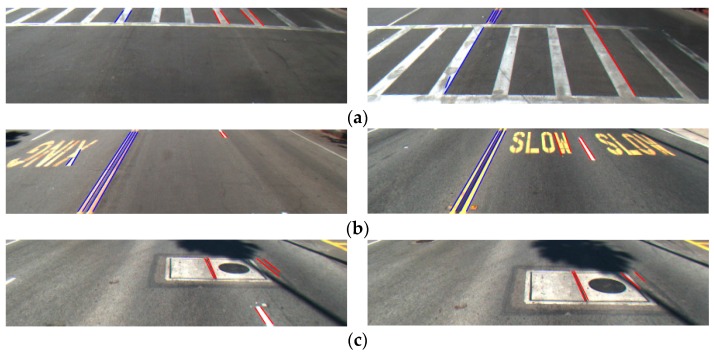
Examples of false detection: (**a**) crosswalk; (**b**) symbol markings of indicating word; and (**c**) non-road objects.

In Figure 12, we show examples of correct detection by our method. Figure 13 shows examples of false detection. As shown in Figure 13a, our method falsely detected the part of a crosswalk on the road as a road lane. In Figure 13b, the part of a text symbol marking is falsely detected as a road lane. In Figure 13c, the boundary of a non-road object is falsely detected as a road lane.

**Figure 14:**
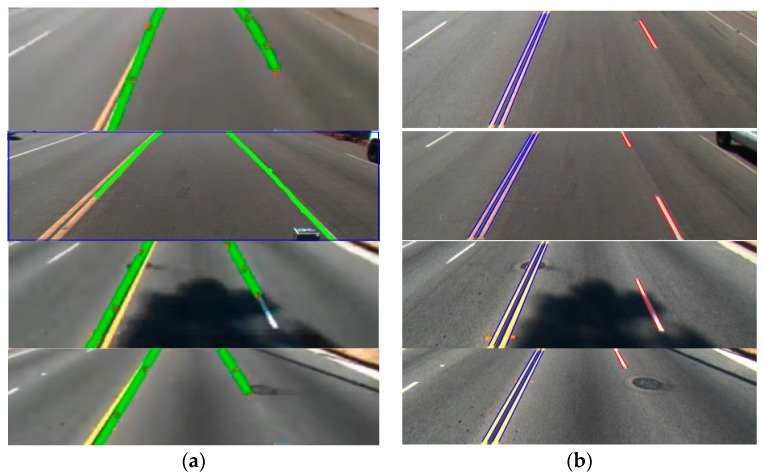
Examples of comparative results: (**a**) Aly’s method [12]; and (**b**) our method.

Next, we compare the performance of our method with that of Aly’s method [12]. Aly used the IPM method to represent a road lane as a straight line in an image, a Gaussian filter and HT to detect straight lines, and RANSAC to fit lane markers. Figure 14 shows the comparisons of lane detection by [12] and our method. The lanes detected by Aly’s method are shown as thick green lines, those detected by our method are represented as blue and red lines. In our comparison, we empirically found the optimal thresholds for both our method and Aly’s method [12], respectively. As shown in Figure 14, Aly’s method cannot accurately discriminate between the dashed and solid lanes. Moreover, his method does not detect the left and right boundaries of each lane.

The reason why our method tries to detect all the boundaries of a road lane is that the type of road lane can be discriminated among two lanes (left blue lanes of Figure 14b) and one lane (right red lane of Figure 14b) by detecting all boundaries. In addition, the reason why our method tries to detect a road lane by discriminating between the dashed and solid lanes is that this discrimination is also important for the driving of an autonomous vehicle.

In Table 3, we compare the lane detection accuracies of Aly’s method [12] and ours, and confirm that our method outperforms Aly’s method [12]. The reason why Aly’s method is less accurate is that Aly’s method cannot accurately discriminate between the dashed and solid lanes as shown in Figure 14. In addition, his method does not detect the left and right boundaries (the red and blue lines of Figure 14b) of each lane. As explained earlier, in our experiment, all the ground-truth positions are marked at all the starting and ending points of the left and right boundaries of dashed and solid lanes. Based on the inter-distance between the two starting positions (ground-truth position and that detected by algorithm) and that between the two ending positions (ground-truth position and that detected by algorithm), whether the detection is successful or has failed is determined. Therefore, Aly’s method gave much higher error rates than our method.

In addition, we included the additional comparative experiments with Truong and coworker’s method [35] with Caltech dataset as shown in Table 3. We empirically found the optimal thresholds for their method, also. As shown in Table 3, our method outperforms Truong and coworker’s method [35], also, because Truong and coworker’s method does not detect the left and right boundaries of each lane with the correct discrimination of dashed and solid lanes, either.

As the next experiment, we measured the processing time per frame by our method as shown in Table 4. The reason why the processing time with the Washington 1 dataset is larger than those with others is that some images of Washington 1 dataset include many shadows which produced many incorrect line segments, and this increases processing time. As shown in Table 4, we can confirm that our method can be operated at a fast speed (about 30.2 frames/sec (1000/33.09)). With all the databases, the processing time for three steps of “Removing the incorrect line segments based on angle”, “Combining two line segments based on inter-distance”, and “Detecting correct lane by the adaptive threshold” of Figure 1 is 0 ms, respectively. Therefore, total processing time is same to that of detecting line segments by LSD algorithm. In future work, we would research about more sophisticated computational techniques to reduce the processing time on the step of detecting line segments by LSD algorithm.

**Figure 15:**
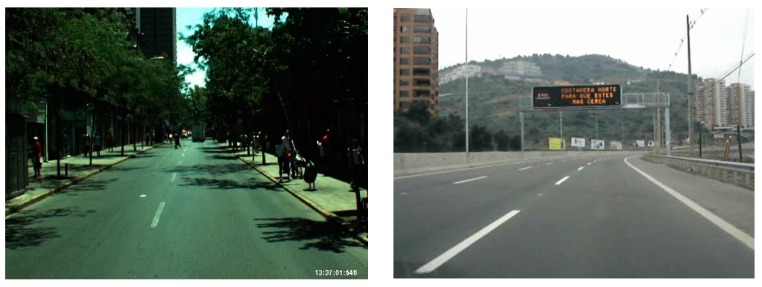
Examples of images on Santiago Lanes Dataset (SLD) datasets.

In addition, we included the comparative experiments by our method with Aly’s method [12] and Truong and coworker’s method [35] with additional datasets of Santiago Lanes Dataset (SLD) dataset [36] as shown in Table 5. The examples of SLD dataset are shown in Figure 15.

**Figure 16:**
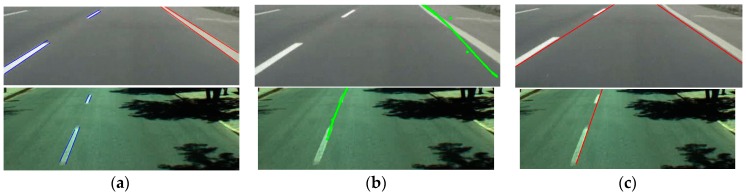
Examples of comparative results of lane detection on SLD datasets: (**a**) our method; (**b**) Aly’s method [12]; and (**c**) Truong and coworker’s method [35].

As shown in Table 5, our method outperforms Aly’s method [12] and Truong and coworker’s method [35] on SLD datasets, also, because their methods do not detect the left and right boundaries of each lane with the correct discrimination of dashed and solid lanes, either, as shown in Figure 16. The examples of detected results on SLD datasets are shown in Figure 16, which shows that our method can detect the starting and ending positions of lane with the discrimination of dashed and solid lanes more accurately than Aly’s method [12] and Truong and coworker’s method [35].

**Figure 17:**
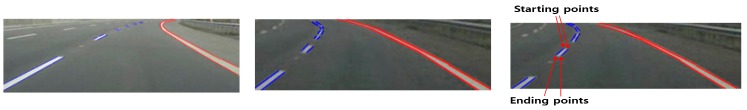
Examples of detection results on curve lanes of SLD datasets by our method (Detected dashed and solid lanes are shown in blue and red colors, respectively).

Our method can correctly detect the curve lane, also. Because Caltech datasets do not include the curve lanes, we include the detection results on SLD datasets by our method. As shown in Figure 17, our method can correctly detect the curve lane, also. In addition, the errors of detection on curve lane are already included in the results of Table 5.

The methods in [31,32] can not only detect lanes, curves as well as straight, but also predict the direction of upcoming curves. However, their method did not detect the starting and ending positions of lane in addition to no discrimination of dashed (broken) and solid (unbroken) road lanes. Different from their methods, our method can correctly detect the starting and ending positions of lane with the discrimination of dashed and solid lanes.

The reason why the accurate detection of starting and ending points is important in our research is as follows. In the case that the lane is changed from dashed lane to solid one, the car should not change its current driving lane. If there exists the error to detect accurate starting and ending points of dashed lane by autonomous car (self-driving car) at fast moving speed, it can causes the traffic accident. For example, assuming that actually dashed lane is ended, but the autonomous car misrecognize the situation as the dashed lane being still maintained due to the error to detect accurate starting and ending points of dashed lane. In this case, the car can change its driving path by crossing of dashed lane (but, actually solid lane). If there exists another vehicle (approaching at very fast speed) behind the autonomous car, and the driver in this vehicle thinks that there would be no lane crossing by the front car (autonomous car) because the dashed lane is ended, the dangerous situation of rear-end collision can happen. These are why we are interest in detecting the accurate starting and ending points of road lane (as shown in Figure 17) in our research.

## 5. Conclusions

In this paper, we presented our research on lane detection, which focuses on how to discriminate between dashed and solid lanes under various environmental conditions. Although it has some limitations in difficult scenarios such as blurred lane markings or shadows, the proposed method shows a stable performance in detecting lanes with images from various environments. All the parameters of our algorithm were empirically determined with some images of dataset without any optimization. We experimentally compared our approach with an existing method and demonstrated the superiority of our method. Because we do not use any tracking information in successive image frames, the detection of lanes by our method is not dependent on the car’s speed. Although our method can detect the correct road lane even with a little amount of shadows, as shown in Figure 12, Figure 14b and Figure 16a, the road lanes with severe amount of shadows cannot be detected due to the limitation of LSD-based detection of line segments.

To overcome the above limitations, we plan to conduct further research on how to reduce the impacts of unexpected noises to enhance the detection accuracy by LSD method and to make the method robust to occlusion. We can also overcome these limitations by using additional classifiers of road symbol markings or indicating text that is written on roads. In addition, the ROI calculation using camera calibration information or the curvature of road markings would be researched in our future work. Furthermore, we would research about the determination of adaptive threshold based on the calibration parameters of camera.

## Figures and Tables

**Table 1:** Comparison of previous and the proposed methods.

Category	Model-Based Method	Feature-Based Method	
		Not Discriminating Dashed and Solid Lanes	Discriminating Dashed and Solid Lanes (Proposed Method)
Methods	- B-spline model [1,2,3,16]. - Hyperbola-pair lane model [5]. - Lane geometrical model [6]. - Vehicle directional control model (DIRCON) [9]. - Quadratic function model [13,15] - IPM model [10,11,12,14,25]. - Linear or parabolic model [17].	- Using edge features [18,22], template correlation [20], EDLines method [21], and illumination invariant lane features [23].	- Detecting the lanes based on line segments. - Incorrect line segments are removed based on the line segments’ angles and by merging the line segments according to their inter-distance. - Using adaptive threshold for detecting the correct lane boundaries.
Advantages	- More accurate results of lane detection can be guaranteed using mathematic models. - Its performance is less affected by noises caused by shadows, water area, and day light.	- Performance is not affected by the initial input parameters of the camera or the model parameters. - Simple and fast processing speed.	- Detecting the accurate left and right boundaries of a road lane. - Discriminating the dashed and solid lanes when detecting the road lanes.
Disadvantages	- The accuracy of the detection depends not only on the initial input parameters of the camera or the shape of the road, but also on the feature points extracted from a captured road image.	- The methods require a clear and strong color contrast of a lane and good road conditions with little effect from changes in the surrounding environment.	- More processing power is necessary for detecting the left and right boundaries of a road lane by discriminating the dashed and solid lanes compared to the methods that only detect the central line of the road lane.
	- They detect the central line of a road lane rather than locating the accurate left and right boundaries of the road lane. - They do not discriminate the dashed and solid lanes when detecting the road lanes.		

**Table 2:** Experimental results with Caltech datasets.

Database	#Images	#TP	#FP	#FN	Precision	Recall	F-Measure
Cordova 1	233	1252	53	92	0.96	0.93	0.94
Cordova 2	253	734	112	15	0.87	0.98	0.92
Washington 1	175	875	94	64	0.9	0.93	0.91
Washington 2	205	1180	196	79	0.86	0.94	0.90
Total	866	4041	455	250	0.90	0.94	0.90

**Table 3:** Comparative accuracies of lane detection on Caltech datasets.

Database Accuracies		Cordova 1	Cordova 2	Washington 1	Washington 2
Precision	Ours	0.96	0.87	0.9	0.86
	[12]	0.012	0.112	0.037	0.028
	[35]	0.553	0.389	0.423	0.440
Recall	Ours	0.93	0.98	0.93	0.94
	[12]	0.006	0.143	0.037	0.026
	[35]	0.512	0.402	0.407	0.430
F-measure	Ours	0.94	0.92	0.91	0.90
	[12]	0.008	0.126	0.037	0.027
	[35]	0.532	0.395	0.415	0.435

**Table 4:** Processing time per each frame (unit: ms).

Module Database	Processing Time
Cordova 1	26.56
Cordova2	32.89
Washington1	37.80
Washington 2	35.12
Average	33.09

**Table 5:** Comparative accuracies of lane detection on SLD datasets.

Database Accuracies		SLD Dataset
Precision	Ours	0.905
	[12]	0.01
	[35]	0.662
Recall	Ours	0.929
	[12]	0.002
	[35]	0.465
F-measure	Ours	0.917
	[12]	0.003
	[35]	0.546
